# American Institute of Homœopathy

**Published:** 1899-09

**Authors:** Benj. F. Bailey

**Affiliations:** President American Institute of Homœopathy; Lincoln, Neb.


### AMERICAN INSTITUTE OF HOMŒOPATHY.

LINCOLN, NEB., August 15th, 1899.

WALTER M. JAMES, M. D., Editor:

The Atlantic City meeting of the American Institute was admittedly one of the greatest and most satisfactory meetings of its history. This was the result of the more thorough appreciation by the profession of its debt to the Institute for the past, and a recognition of the possibilities for the future.

No business prospers that is only furthered by periodical spasms of interest. There is a necessity for watchful, persistent work twelve months in the year. The business of the American Institute is no exception to this rule. The present officers of the Institute desire to be faithful to their trust to the very last minute of their tenure of office, that they may be able to place the Institute in the care of their successors strong and well equipped. Now, as in the earlier part of the year, this is only possible by the faithful help of the individual members. During the remaining four or five months of 1899 the canvass for new members should continue, each member being loyal enough to determine to secure at least one application for membership. This can easily be done, and we appeal to the Institute membership to give their attention to this promptly.

Application blanks may be secured of the Secretary, Dr. Eugene H. Porter, 181 West 73d Street, New York City, New York, and when filled should be sent with the necessary seven dollars to Dr. Geo. B. Peck, Providence, R. I., Chairman of the Board of Censors.

And yet, after all, what doth it profit a society if we enlist new blood only to lose each year nearly as many who have only joined from chance or some circumstance of social interest? We appeal to the "old guard" to stand firm, not merely retaining their membership, but keeping in close touch with the officers and committees of the Institute and doing continual missionary and organization work in their respective localities. We want every present member of the Institute to remain in the work. We want applications and fees for five hundred new members in the hands of the Board of Censors by January 1st, 1900. So easily done, if each one does his duty!

To foster this work we request that those who have been faithful members of the Institute give to the medical press in a few words the reason for their faith and loyalty. We are sure that the journals will be more than glad to give space for hundreds of such twenty or thirty-word letters. You love the old Institute,—tell your fellow why! Arouse his interest, push the work along. *Don't wait.* Write that word *at once.*

This is a work the body of the Institute can prosecute. That the committees will vigorously execute the detail of committee work we have no question; but there is a work resting in the hands of one committee that cannot be carried to successful completion without the aid and abetment of the individual,—this is the work of the Hahnemann Monument Committee. A monument is already completed and ready for erection in Washington, that noble Capitol City of our land. A work of art second to none; when erected, a constant reminder to an ever-passing public of our honor and gratitude to the father of our faith; an argument stronger than words for the strength of our school, and such an example of art that he who sees cannot forget; and remembering, he thinks again and gratefully of you, of me, of all of that school of medicine that through their love and loyalty have given such a gift of love to the people.

Would we be known and respected throughout the length and breadth of the land, we must write our history and work on the public scrolls. This committee, under the direction of the American Institute and the encouragement of the profession, contracted for this work. The Monument Committee has done its work and done it well, and now asks you who gave them your work to do for the necessary amount to meet their liabilities so that this monument may be erected free from debt early in 1900.

The committee will make a most vigorous canvass during the fall—the money must be raised—it will be raised. We know enough of the personnel of our profession to rest assured that early, yes, easy, response and success awaits the work of this committee.

Proud of your inheritance, anxious for the perpetuity of the memory of your benefactor in the faith, lay aside something for this work.

Fraternally,

BENJ. F. BAILEY,
*President American Institute of Homœopathy.*

